# Vericiguat: The Fifth Harmony of Heart Failure with Reduced Ejection Fraction

**DOI:** 10.3390/jcdd10090388

**Published:** 2023-09-08

**Authors:** Luigi Falco, Benedetta Brescia, Dario Catapano, Maria Luigia Martucci, Fabio Valente, Rita Gravino, Carla Contaldi, Giuseppe Pacileo, Daniele Masarone

**Affiliations:** 1 Heart Failure Unit, Department of Cardiology, AORN dei Colli-Monaldi Hospital, 80131 Naples, Italy; luigifalco94@libero.it (L.F.); dariocat90@gmail.com (D.C.); marilumartucci93@gmail.com (M.L.M.); dr.valentefabio@gmail.com (F.V.); rita.gravino@ospedalideicolli.it (R.G.); contaldi.carla@gmail.com (C.C.); gpacileo58@gmail.com (G.P.); 2 Department of Experimental Medicine, University of Campania “Luigi Vanvitelli”, 80131 Naples, Italy; benedettabrescia@libero.it

**Keywords:** heart failure with reduced ejection fraction, worsening heart failure, vericiguat

## Abstract

Heart failure with reduced ejection fraction is a chronic and progressive syndrome that continues to be a substantial financial burden for health systems in Western countries. Despite remarkable advances in pharmacologic and device-based therapy over the last few years, patients with heart failure with reduced ejection fraction have a high residual risk of adverse outcomes, even when treated with optimal guideline-directed medical therapy and in a clinically stable state. Worsening heart failure episodes represent a critical event in the heart failure trajectory, carrying high residual risk at discharge and dismal short- or long-term prognosis. Recently, vericiguat, a soluble guanylate cyclase stimulator, has been proposed as a novel drug whose use is already associated with a reduction in heart failure-related hospitalizations in patients in guideline-directed medical therapy. In this review, we summarized the pathophysiology of the nitric oxide-soluble guanylate cyclase-cyclic guanosine monophosphate cascade in patients with heart failure with reduced ejection fraction, the pharmacology of vericiguat as well as the evidence regarding their use in patients with HFrEF. Finally, tips and tricks for its use in standard clinical practice are provided.

## 1. Introduction

Heart failure with reduced ejection fraction (HFrEF) represents an increasingly significant global health concern, which imposes substantial economic costs on Western countries due to high hospitalization rates, morbidity and mortality [1]. Despite significant improvements in drug [2,3] and device-based therapy [4] occurring in recent years, approximately 20–25% of HFrEF patients present phases of worsening clinical symptoms (particularly those related to congestion) with the need for increased doses of diuretics or intravenous diuretic therapy [5]. This stage of the disease is called worsening heart failure (WHF) and denotes disease progression and a potentially poor prognosis for the patient [6].

Therefore, novel therapeutic approaches are needed to reduce WHF in patients in guideline-directed medical therapy (GDMT). Recently, vericiguat, a soluble guanylate cyclase (sGC) stimulator, was shown in randomized clinical trials to reduce cardiovascular events and WHF episodes in HFrEF patients in GDMT [7]. In this review, the rationale for the use of vericiguat, as well as the clinical evidence for their use in clinical practice, were summarized.

## 2. Pathophysiology of Nitric Oxide-sGC-Cyclic Guanosine Monophosphate Cascade in HFrEF

**Figure 1:**
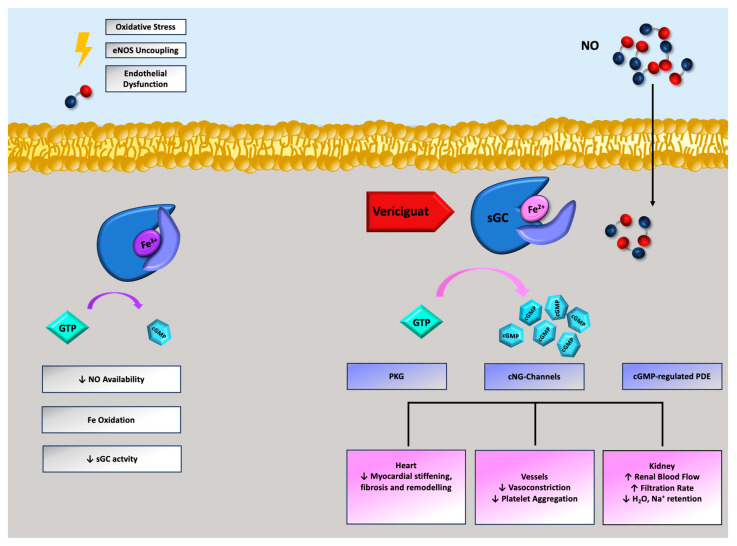
Biological effects of NO-sGC-cGMP cascade and vericiguat’s role. NO: nitric oxide; sGC: soluble guanylate cyclase; cGMP: cyclic guanosine monophosphate; PKG: cyclic GMP-dependent protein kinase; cNG: cyclic nucleotide-gated; PDE: phosphodiesterase.

The nitric oxide (NO)-sGC-cyclic guanosine monophosphate (cGMP) cascade plays a crucial role in cardiovascular homeostasis [8]. The primary source of NO is the endothelium, which is continuously stimulated by hormones and physical triggers to produce it through endothelial NO synthase (eNOS) activity [9,10]. NO is highly diffusible, and thus, quickly spreads into smooth muscle cells, increasing the activity of sGC and, therefore, the production of cGMP, which exerts several positive effects in multiple organs [11] (Figure 1). In HFrEF, the presence of low-grade but persistent inflammation and endothelial dysfunction results in decreased bioavailability of NO, leading to reduced cGMP synthesis [12]. A deficiency of cGMP causes coronary and renal circulatory dysfunction, as well as microcirculatory dysfunction, perpetuating a vicious cycle of myocardial injury and fibrosis with further inflammation [13,14,15]. Given the crucial role that cGMP plays in the pathophysiology of HFrEF (Figure 1), recent research has focused on the search for drugs that can interact in a “smart” manner [16,17] with this pathophysiological cascade, unlike “traditional” drugs, such as nitrates and phosphodiesterase inhibitors [18,19]. Activators of sGCs, such as cinaciguat, increase cGMP levels through direct (NO-independent) activation of sGCs, and are theoretically helpful in patients with acute heart failure or advanced heart failure (and therefore with a greater degree of reduction in NO levels related to oxidative stress and endothelial dysfunction); however, their clinical use is limited by symptomatic hypotension [20,21]. In contrast, sGC stimulators (riociguat and vericiguat), by increasing the sensitivity of sGC to endogenous NO, exert a more physiologic action, which, while causing clinical benefits to be absent in the advanced stages of the disease (see below), justifies the lower rate of symptomatic hypotension [22,23].

## 3. Clinical Pharmacology of Vericiguat

Vericiguat belongs to the class of sGC-stimulating drugs like riociguat, the first of the drugs in this class to have been approved for clinical use in patients with pulmonary arterial hypertension (PAH) [24]. Vericiguat differs from riociguat because it has a longer half-life, allowing it to be administered orally once daily. Vericiguat does not show inhibitory effects on main cytochrome (CYP) isoforms. Indeed, it undergoes a phase II metabolic pathway provided by glucuronic acid conjugation. Excretion is balanced between urine and feces [25,26]. Several phase I studies in healthy subjects assessed the pharmacokinetic profile of vericiguat, and only a few interactions were demonstrated. None of these included medications commonly prescribed in HFrEF patients. However, the concomitant use of nitrates or PDE inhibitors is not recommended because of the potential synergic effect on vasodilation and, therefore, the risk of symptomatic hypotension [27]. Vericiguat acts as a nitrovasodilator and acts synergistically with endogenous NO by stimulating sGCs and promoting cGMP production even under conditions of low NO levels, but unlike other nitrovasodilators, it does not induce long-term tolerance [28,29]. The effects mediated by vericiguat on the NO-sGC-cGMP pathway in patients with HFrEF are varied and include reduced vascular resistance with decreased afterload of both ventricles [28] and improvement in renal and coronary perfusion [30]. In addition, increased intracytoplasmic cGMP concentrations result in increased titin phosphorylation (due to increased protein kinase G activity), improved diastolic function, and increased cardiac index [31,32]. Among the extracardiac effects beneficial in patients with HFrEF, the improvement in renal function and the reduction in kidney fibrosis [33] seem to be the most important.

## 4. Clinical Evidence Supporting the Use of Vericiguat in HFrEF

The use of vericiguat in humans was initially conducted in phase 1 studies in healthy volunteers between 2011 and 2015; these studies essentially demonstrated good tolerability of the drug at dosages no higher than 10 mg and that it has less variable pharmacokinetics when taken with food. From 2015 onward, phase 2 and phase 3 studies began in patients with HFrEF.

### 4.1. SOCRATES-Reduced

SOCRATES-Reduced (Effect of Vericiguat, a Soluble Guanylate Cyclase Stimulator, on Natriuretic Peptide Levels in Patients with Worsening Chronic Heart Failure and Reduced Ejection Fraction), a phase II dose-finding RCT, evaluated four different dose regimens of vericiguat in HFrEF patients with a WHF event within four weeks from randomization [34]. The eligibility criteria included a history of chronic HF with left ventricular ejection fraction (LVEF) < 45%, NYHA class II–IV, a standard background of HF therapy, and clinical stabilization after a WHF event. WHF was defined as worsening of clinical status requiring admission or outpatient diuretics with signs and symptoms of congestion. Patients were deemed stable after withdrawal of i.v. vasodilators for >24 h and i.v. diuretics for >12 h. Additionally, systolic blood pressure > 110 mmHg and heart rate > 50 bpm were required. The biochemical inclusion criteria were B-type natriuretic peptide (BNP) levels > 300 pg/mL for patients with sinus rhythm or >500 pg/mL for atrial fibrillation patients. If the NT-proBNP levels were used for enrollment, the cutoffs were >1000 or >1600 pg/mL for sinus rhythm and atrial fibrillation, respectively. After an initial screening phase, patients were randomized to one of the four vericiguat groups or the placebo group. The study failed to reach the primary endpoint, i.e., the change in log-transformed NT-proBNP, from baseline to week 12 [35]. No difference was seen in log-transformed NT-proBNP plasma levels between the pooled vericiguat experimental group (1.25 mg, 2.5 mg, 5 mg and 10 mg once daily) and the placebo group (*p* = 0.15). Considering individual vericiguat groups, though, 10 mg vericiguat significantly decreased NT-proBNP levels compared to the placebo (*p* = 0.48). Moreover, an explorative secondary analysis using a linear regression model found a significant reduction in NT-proBNP with higher doses of vericiguat (*p* = 0.02). Although the study was not powered to assess clinical endpoints, after three months, the incidence of WHF was 9.9% in the vericiguat group compared with 17.4% in the placebo group. The highest-dose group had 11% of a composite rate of cardiovascular mortality or WHF, whereas the placebo group had 19.6%. Besides lower NT-proBNP levels, improved LVEF (*p* = 0.02) was reported in the 10 mg vericiguat group without changes either in blood pressure or heart rate, suggesting that vericiguat induces left ventricular reverse remodeling without inducing symptomatic hypotension. Overall, vericiguat had a good safety profile. Hypotension and syncope were more common in the 10 mg vericiguat group. However, most of these adverse events (AEs) occurred in the first two weeks when, according to the titration schedule, patients were taking a low dose (2.5 mg), which did not lead to unacceptable withdrawal rates. This investigation generated the idea that a once-daily sGC stimulator, when used with current medications, may safely exert beneficial cardiac effects. This would be in line with the preclinical data for sGC modulators in HF, which have shown that these agents have been associated with blood pressure-independent reductions in cardiac fibrosis and left ventricular mass, improvements in endothelial dysfunction, and the preservation of renal function.

### 4.2. VICTORIA Trial

The VICTORIA (Vericiguat Global Study in Subjects with Heart Failure with Reduced Ejection Fraction) study was a multinational, double-blind, placebo-controlled phase III RCT [36] that expanded the information gathered in SOCRATES-Reduced. The study enrolled 5050 patients with LVEF < 45%, elevated natriuretic peptide levels according to rhythm (BNP > 300 pg/mL or NT-proBNP > 1000 pg/mL in sinus rhythm and BNP > 500 pg/mL or NT-proBNP > 1600 pg/mL in patients with atrial fibrillation), and recent WHF. WHF was defined as prior hospitalization within six months from randomization, or outpatients requiring i.v. diuretics within three months. Furthermore, the systolic blood pressure exclusion cutoff was decreased from 110 to 100 mmHg since vericiguat did not have any appreciable impact on blood pressure in the SOCRATES-Reduced study. A 30-day screening phase was followed by 1:1 randomization to vericiguat or the placebo. Participants were treated in an event-driven fashion until the required number of events occurred. Despite an estimated follow-up of 18 months, the median follow-up duration was less than a year (10.8 months) due to the higher event rate that reflected the high risk of the cohort. Indeed, 43% of patients had an estimated glomerular filtration rate of less than 60 mL/min/m^2^. Even lower renal function (eGFR < 30 mL/min/m^2^) was found in 10% of the population. The primary endpoint was a composite of death from cardiovascular causes and HF-related hospitalizations. Importantly, the Food and Drug Administration (FDA) requested that authors pre-specify the quartiles of baseline natriuretic peptides levels. Despite a very high placebo event rate (38% over 12 months), the primary endpoint was reached (HR: 0.90; 95% CI: 0.82–0.98; *p* = 0.02). This equates to a 4.2% annualized absolute risk reduction with a number needed to treat of 24 patients, which is rather appealing. Analyzing the components of the primary endpoint, only HF-related hospitalizations were significantly reduced by vericiguat when accounting for statistical significance of the primary outcome. Additionally, among the secondary endpoints, the composite of death from any cause or first HF hospitalization reached statistical significance (HR: 0.90; 95% CI: 0.83–0.98; *p* = 0.02.) The median level of NT-proBNP was 2816 pg/mL, nearly doubling NT-proBNP levels in previous pivotal HFrEF RCTs (PARADIGM-HF: 1615 pg/mL [37], DAPA-HF: 1437 pg/mL [38]). Interestingly, a significant interaction emerged between NT-proBNP quartiles and the primary endpoint. This finding was further explored by Ezekowitz et al. [39]. Vericiguat efficacy was maintained until NT-proBNP levels of 8000 pg/mL, which correspond to 86% of the population (adjusted HR: 0.85; 95% CI: 0.76–0.95 for primary endpoint; adjusted HR: 0.84; 95% CI 0.71–0.99 for cardiovascular death; adjusted HR: 0.84; 95% CI: 0.75–0.95 for HF-related hospitalizations). The benefits were even greater among participants with NT-proBNP < 4000 pg/mL (adjusted HR: 0.77; 95% CI: 0.68–0.88 for primary endpoint; adjusted HR: 0.75; 95% CI: 0.60–0.94 for cardiovascular death; adjusted HR: 0.78; 95% CI: 0.67–0.90 for HF-related hospitalizations). Therefore, the authors identified a non-linear relationship between NT-proBNP levels and clinical outcomes, with a more pronounced rise over 4000 pg/mL. As could be expected, a closer examination of the baseline characteristics of the 15%–16% of patients whose NT-proBNP levels exceeded 8000 pg/mL reveals that they were older, had a more advanced functional NYHA class and had a higher prevalence of renal and other comorbidities. The discrepancies in efficacy and safety outcomes according to NT-proBNP levels at randomization were also assessed in a more recent post hoc analysis [40] comparing patients in the first three quartiles (Q1–Q3, NT-proBNP: <1556 pg/mL–>2816–5314 pg/mL), evaluated as a single group, to those in the upper fourth quartile (Q4 NT-proBNP: >5314 pg/mL). Similarly to the analysis presented above, Senni et al. [40] found that Q4 patients had several features of more advanced disease. Indeed, they were older and were more likely to have LVEF < 40% and eGFR < 30 mL/min. They had lower mean LVEF, eGFR, hematocrit and hemoglobin. More patients in Q4 compared to Q1–Q3 patients were classified as NYHA class III and class IV and had an increased MAGGIC risk score. Atrial fibrillation was more frequent in Q4 patients. Background GDMT was lower (triple therapy in Q4 51.3% vs. 61.4% in Q1–Q3). In addition, vericiguat was underdosed and more frequently discontinued. Finally, more patients in Q4 were enrolled at hospital admission or had a history of HF-related hospitalization within three months. These high-risk features, overall, may account for higher event rates in the vericiguat arm compared to the placebo. Indeed, Q4 patients taking vericiguat were more likely to experience the primary outcome (57.6% vs. 51.6%, HR: 1.15; 95% CI: 0.99–1.34; *p* = 0.070). Moreover, cardiovascular death and HF-related hospitalizations occurred in 33.8% (HR: 1.16; 95% CI 0.95–1.43; *p* = 0.145) and 40.7% (HR: 1.19; 95% CI 0.99–1.44; *p* = 0.058) of patients, respectively, in the vericiguat group compared to 28.9% and 35.7% in patients taking the placebo. These results contrast with the findings provided by Ezekowitz et al. [39], demonstrating persistence of benefits until NT-proBNP levels of 8000 pg/mL compared to 5314 pg/mL. However, despite differences in the NT-proBNP cutoffs, a lack of benefit for more advanced patients emerged. Based on the SOCRATES-Reduced findings, the target dose of vericiguat was 10 mg. Adherence was not an issue; indeed, 90% of patients were on the target dose of vericiguat. Moreover, despite prespecified AEs of clinical interest being more frequent among participants taking vericiguat, neither symptomatic hypotension (9.1% vs. 7.9%) nor syncope (4% vs. 3.5%) achieved statistical significance (*p* = 0.12 and *p* = 0.3, respectively). Other AEs included gastrointestinal disorders such as diarrhea (5.2% vs. 4.9%), dyspepsia (2.7% vs. 1.1%), nausea (3.8% vs. 2.7%), vomiting (2.2 vs. 1.8%), ear and labyrinth disorders (2.7% vs. 2.1%), influenza (2.9% vs. 2.3%), hypotension (15.4% vs. 14.1%), dizziness (6.7% vs. 6.0%), headache (3.4 vs. 2.4%), acute kidney injury (5.3% vs. 5.0%), renal failure (3.7% vs. 3.5%), renal impairment (2.7% vs. 2.6%), hypokalemia (3.7% vs. 3.5%), hyperuricemia (3.1% vs. 2.9%), COPD (3.0% vs. 2.3%), cough (4.4% vs. 4.2%) and dyspnea (5.3% vs. 5.1%). VICTORIA patients assigned to receive vericiguat had a greater rate of anemia and slightly lower hemoglobin 16 weeks after the start of treatment [41]. Anemia is a common comorbidity of HF, and it is related to poor outcomes [42]. Consistent data were reported in patients enrolled in VICTORIA. Previous animal studies have highlighted a link between the sGC pathway and hemoglobin [6]. Moreover, in an RCT evaluating riociguat for the treatment of PAH, an association with anemia emerged [43]. The underlying pathophysiology, though, is still unclear. Participants taking vericiguat were more likely to report anemia-related AEs than those taking a placebo (7.6% vs. 5.7%). After four weeks of treatment, they had a greater fall in Hb levels. This difference was persistent throughout the study. Additionally, emergent anemia was more common in the vericiguat group. Nevertheless, these findings did not have any impact on vericiguat efficacy. Atrial fibrillation was common in this high-risk population and was linked to higher rates of cardiovascular death either when diagnosed before or post-randomization. Unlike the previous post hoc analysis focusing on the progressive decline in hemoglobin, new-onset atrial fibrillation was equally distributed between the placebo and vericiguat groups. Moreover, vericiguat’s effects on the primary composite endpoint were not affected by the arrhythmic status of patients [44]. Nearly 15% of patients in VICTORIA were taking an ARNI. Despite a lower LVEF and a higher use of GDMT and device therapy, sacubitril/valsartan use did not significantly interact with vericiguat’s beneficial effects [45]. Considering the shared downstream effects of sacubitril/valsartan and vericiguat, as well as potential common AEs the next step is assessing the efficacy and safety of the concomitant use of both medications. The authors of VICTORIA should be congratulated for conducting the first-ever evaluation of vericiguat interaction in ARNI implementation. On one hand, more patients in the placebo group started sacubitril/valsartan compared to those taking vericiguat. This difference occurred early and broadened throughout the study course. On the other hand, among participants already taking sacubitril/valsartan, the withdrawal rate was similar regardless of the treatment group. The prognosis for HF patients with coronary artery disease (CAD) is poor. Indeed, the incidence of both cardiovascular death and WHF was greater in individuals with CAD compared to those without CAD. Vericiguat was shown to be just as safe in this group as was originally thought, with fewer cases of syncope and clinical hypotension [46]. It is unknown why vericiguat does not have a more dramatic therapeutic impact in individuals with HFrEF and concurrent CAD, given the expected involvement of reduced sGC activity in endothelial dysfunction and microvascular illness. However, since myocardial infarction and stroke are very uncommon, it would be challenging to show a treatment’ s positive impact on these tertiary outcomes due to their low prevalence. The very short follow-up duration of 10.8 months (motivated by high event rates) may have possibly masked a treatment benefit that would become apparent with more observation.

The improvement of quality of life (QoL) is a major goal in HFrEF management; therefore, QoL assessment using valid questionnaires has become mandatory when new HF medications are evaluated. Butler et al. [47] did not only study vericiguat’s effects on health status using the Kansas City Cardiomyopathy Questionnaire (KCCQ), but investigated the potential interaction between baseline health status and clinical outcomes. KCCQ was administered to patients at baseline and at 1 month, 4 months and 8 months after randomization. Three tertiles were identified based on KCCQ scores. Improvements in QoL throughout the study were similar in both groups (interaction *p* = 0.97). Similar results were found upon analyzing patients according to WHF event, except for a trend among patients with NT-proBNP levels of 4000–8000 pg/mL. On the other hand, the incidence of a primary endpoint was reduced with vericiguat regardless of health status (interaction *p* = 0.13). These findings suggest that even if vericiguat did not provide any added value in terms of QoL, the beneficial effects of vericiguat are consistent across the spectrum of baseline clinical status. 

VICTORIA was designed with the aim to enroll a broad HFrEF population after a WHF at least 6 months before randomization. Increasing focus has been given to WHF, with worldwide attempts to further characterize the risk and prognosis of both HF-related hospitalizations and clinical deterioration treated with an outpatient intensification of diuretic therapy. [5,6,48] Originally, the prognosis was considered similar; however, recent evidence suggests a higher risk of hospitalizations. A post hoc analysis of VICTORIA was consistent with the latest evidence [49]. More than half of the VICTORIA population was enrolled within three months after the index HF-related hospitalization, 17% of patients were hospitalized between three and six months and 16% were outpatients. There was a trend towards greater risk as close as the index event with respect to randomization. Additionally, among patients with an index event within three months, those hospitalized had a greater incidence of primary composite endpoint (40.9 events per 100 patient-years vs. 23.4 events per 100 patient-years, adjusted HR: 1.48; 95% CI: 1.27–1.73). The risk was the highest among patients recruited at admission (>50 events per 100 patient-years). Nevertheless, an interaction between treatment effects and was not detected (interaction *p* = 0.09). Including a broad population in HF RCTs is important to easily translate the results achieved in a controlled environment to the real world where comorbidities can affect tolerability and uptitration. Lam et al. [23] identified hypotension-vulnerable phenotypes across the VICTORIA population and evaluated the safety and efficacy for each subgroup. The following features were common in vulnerable patients: age > 75 years (27.6%), systolic blood pressure between 100 and 110 mmHg (26.6%) and treatment with ARNI (14.5%). As expected, older patients had a greater decline in systolic blood pressure. However, after four months of treatment, there was a similar trend towards baseline systolic blood pressure regardless of age. Interestingly, patients with SBP < 110 mmHg, did not show further blood pressure reduction, on the contrary SBP increased from the first week. The occurrence of AEs, such as hypotension and syncope, was related to a higher incidence of primary outcomes (adjusted HR: 1.55; 95% CI: 1.31–1.82). However, AE rates were not significantly different between the placebo and vericiguat groups, both considering data before and after the titration phase (respectively, adjusted HR: 1.18; 95% CI: 0.99–1.39; *p* = 0.059 and HR: 1.13; 95% CI: 0.93–1.37; *p* = 0.22). Finally, efficacy was consistent across subgroups (interaction *p* = 0.32). 

## 5. Place of Vericiguat

Although previous attempts at targeting NO gave conflicting results [50,51,52,53], vericiguat successfully leverages the NO-sGC-cGMP pathway, finally translating a well-known biochemical rationale to improved clinical outcomes. However, these results arrive in a scenario dominated by the recent groundbreaking results of ARNI [37] and SGLT2 inhibitors [38,54], risking overshadowing vericiguat’s added benefits. This risk is potentially enhanced by misleading interpretation of the VICTORIA results. Physicians tend to focus on HRs when analyzing RCTs; however, they should be cautious and be aware of differences among studies. Indeed, even a small relative risk reduction in a cohort with a considerably high rate of events translates into a significant absolute risk reduction. This is precisely the case for VICTORIA, in which the event rates nearly tripled HF hospitalizations and cardiovascular death compared to PARADIGM-HF and DAPA-HF (Table 1). 

Once vericiguat’s benefits have been clarified, the next step is finding the right spot for vericiguat in the enviable landscape of HFrEF medications. The benefit of quadruple therapy at recommended dosages is well established [55]. Less clear is how to initiate and titrate foundational therapies. A rigorous sequential therapy is a slow process that often limits the achievement of target doses and benefits. Indeed, contemporary registries highlight poor rates of GDMT [56,57,58], especially for triple therapy and target doses. Several strategies have been proposed by experts for the rapid initiation of GDMT [59,60,61]. However, clinicians may fear increased rates of AEs and complain about a lack of robust evidence. Recently, the STRONG-HF RCT has provided solid data supporting the efficacy, safety and feasibility of intensive and rapid therapy optimization [62]. Indeed, the primary outcome of the study, a composite of all-cause death and HF hospitalizations at 6 months from randomization, reached statistical significance (*p* = 0.002). Nevertheless, EMPEROR-Reduced [54] reported significant residual all-cause death and HF hospitalizations (20% and 19%, respectively) despite quadruple therapy in the empagliflozin group, suggesting that there is still room for improvement. However, since one in three patients was not receiving triple therapy and only a few patients in VICTORIA were taking an SGLT2 inhibitor, further studies are needed to elucidate the impact of vericiguat on residual risk. On the other hand, despite the early skepticism about the low relative risk reduction provided by vericiguat, the VICTORIA findings have been rapidly adopted by international societies [63,64,65] (Table 2). Indeed, the worldwide updated HF guidelines recommend vericiguat as additional therapy, in addition to GDMT, in high-risk patients who have recently recovered from WHF, to reduce cardiovascular death and HF hospitalizations. However, a closer look reveals a few limitations of the current recommendations. Indeed, the European guidelines recommend vericiguat initiation in NYHA class II-IV patients experiencing WHF despite triple therapy (i.e., ACE inhibitors/ARB/ARNI, beta blocker and MRA) without mentioning SGLT2 inhibitors [63]. The Canadian and American guidelines advise starting vericiguat when patients are already taking GDMT [64,65]. In addition, the recently published “Focused Update of the 2021 ESC Guidelines for the diagnosis and treatment of acute and chronic heart failure” [66] does not provide any new suggestions on vericiguat. Collectively, the recommendations on vericiguat use remain quite elusive, and further studies to better phenotype patients who are going to benefit from vericiguat are needed. Nevertheless, safety data and post hoc analyses suggest that vericiguat could be easily incorporated into the existing four pillars, providing a fifth option addressing a long-term unmet needs of patients with WHF. Efficacy and safety, though, need to be matched with costs. Indeed, cost-effective medications are crucial in HF care as the incidence increases worldwide, along with the economic burden, mainly owing to hospitalizations and direct medical expenditures. A recent analysis has shown the positive cost-effectiveness ratio of vericiguat, despite its high cost [67]. When added to routine therapy for individuals with chronic heart failure after a deteriorating episode, vericiguat did not show an important budget impact due to reductions [68]. However, the lack of long-term data and the paucity of information about discontinuation frequencies, AEs and event rates limit the real-world generalizability of this analysis.

Currently, we advise starting vericiguat on top of HFrEF’s four pillars in patients who fulfil the eligibility criteria of VICTORIA. Since vericiguat boosts an endogenous pathway acting as an agonist, wash-out periods and withdrawals are not required. Uptitration should follow the RCT protocol, adjusting the doses every two weeks. Further dose adjustments based on renal and liver function are not necessary. Indeed, VICTORIA set the lowest cutoff of eGFR for a HFrEF RCTs to a striking 15 mL/min/m^2^ (eGFR inclusion criteria of PARADIGM-HF and DAPA-HF >30 mL/min/m^2^, EMPEROR-Reduced > 20 mL/min/m^2^). Not only were the benefits consistent irrespective of renal function, but the eGFR slope was the same between the vericiguat and placebo groups [69]. Healthcare providers do not have to worry about hypotension or electrolyte disturbances, enabling concomitant therapy with ACE inhibitors, ARB, ARNI and MRA. Conversely, anemia was more frequent with vericiguat. Therefore, periodical biochemical monitoring, including complete blood count, could be useful, although not imperative. Currently, a single RCT in a high-risk population with limited follow-up is available for vericiguat in HFrEF. Thus, the careful profiling of HFrEF patients, as suggested by a recent ESC/HFA position paper [70], is pivotal to ensure clinical benefit. Lam et al. [49] have shown that vericiguat’s benefits cover the entire spectrum of WHF. Therefore, a deteriorating event is the cornerstone of vericiguat initiation. A patient on maximally tolerated GDMT suddenly experiencing worsening of dyspnea, weight gain and a rise in proBNP-NT levels who requires diuretic therapy intensification in an outpatient regimen is a suitable candidate for vericiguat. Similarly, a patient previously admitted for WHF could start vericiguat to further reduce the risk of subsequent HF-related hospitalizations, especially in patients with limited therapeutic options, such as those with concomitant severe CKD. Indeed, these patients cannot achieve quadruple therapy since RAAS inhibitors are contraindicated with eGFR < 30 mL/min/m^2^. Hospitalized patients, once stabilized, also become suitable candidates. Initiation may be considered either in hospital or in the early post-discharge period without prolonging hospital stay. In this setting vericiguat’s benefits might not be limited to reduced HF-related hospitalizations, and it could work as enabler medication for beta-blockers, especially in patients with significant residual congestion [70]. Finally, a thorough evaluation of clinical high-risk features and NT-proBNP assessment before starting vericiguat is crucial regardless of the clinical scenario, as seminal post hoc analyses of VICTORIA have questioned vericiguat’s benefits in patients with more advanced disease [39,40].

## 6. Real-World Experience

A simultaneous, parallel, multicenter observational registry was conducted in the same centers that conducted the VICTORIA trial [71]. Data were collected retrospectively, and potential reasons for the lack of trial participation were identified. Concomitant use of nitrates and low blood pressure were the most common causes of ineligibility. However, nearly 40% of patients fulfilled the RCT inclusion criteria, suggesting that the VICTORIA cohort was representative of real-world HFrEF patients. The Practice Innovation and Clinical Excellence (PINNACLE) registry aimed to describe the epidemiology, clinical features, medications used and outcomes of the patients enrolled [72]. Unique attention was given to treatment patterns before WHF, at hospital admission and post-discharge. A first evaluation of the real-world result generalizability of VICTORIA was conducted by analyzing the PINNACLE data [73]. About a quarter of the PINNACLE population fulfilled the WHF criteria used in the VICTORIA trial. Patients in the RCT shared several clinical and laboratory features with registry patients who had experimented with WHF. The latter had worse outcomes compared to those without a WHF event. However, few notable differences were found. On one hand, VICTORIA patients had a higher NYHA class. On the other hand, they had higher systolic blood pressure and higher rates of GDMT. These findings may account for better safety and outcomes, respectively. Indeed, the VICTORIA placebo group had better outcomes compared to PINNACLE patients. Two different studies have evaluated whether enrolled patients fulfilled not only VICTORIA’s inclusion criteria but also the FDA label criteria [74,75]. In the Korean Acute Heart Failure (KorAHF) registry [74], lower systolic blood pressure and NT-proBNP levels were the leading reasons for not fulfilling the inclusion criteria of the VICTORIA trial. In addition, the label criteria were met more often than VICTORIA criteria. Nguyen et al. [75] reported similar results upon analyzing HFrEF patients from SwedeHF, suggesting that the design of VICTORIA successfully recruited high-risk patients at the cost of limiting real-world eligibility. Nevertheless, all currently approved HF medications have been tested in both low- and high-risk patients [76]. The greater benefit seen in patients with lower NT-proBNP levels and in those with a longer duration between index HF hospitalization and randomization suggest the potential to improve outcomes even in lower-risk, stable patients. Indeed, a phase III RCT, A Study of Vericiguat (MK-1242) in Participants with Chronic Heart Failure with Reduced Ejection Fraction (HFrEF) (MK-1242-035) (VICTOR) is ongoing (https://classic.clinicaltrials.gov/ct2/show/NCT05093933?term=mk-1242&draw=2) (accessed on 6 August 2023), randomly assigning chronic HFrEF participants who have not had recent WHF to receive either vericiguat or a placebo on top of standard of care.

## 7. Conclusions

Vericiguat is the latest drug added to the therapeutic landscape of HFrEF. Based on the VICTORIA trial’s findings, vericiguat is currently recommended by both the European and American HF guidelines for HFrEF patients with recent WHF. While the clinical and prognostic relevance of WHF is increasingly being recognized, vericiguat successfully addresses an important unmet need, reducing the incidence of cardiovascular death and WHF in high-risk HFrEF patients. However, event reduction was reported only in patients with NT-proBNP baseline levels in the three lower quartiles and up to 8000 pg/mL, suggesting major benefits in less advanced HFrEF. Further studies are warranted to find the right application for vericiguat. Hopefully, VICTOR will provide further insights into the management of ambulatory patients with clinically stable HFrEF.

## Figures and Tables

**Table 1:** Comparison of main features of PARADIGM-HF, DAPA-HF, and VICTORIA.

Trial	PARADIGM-HF [37]	DAPA-HF [38]	VICTORIA [36]
Year	2014	2019	2020
Mean Age, years	63.8	66.3	67.3
Mean LVEF, %	29	31.2	29
Median NT-proBNP, pg/mL	1631	1428	2816
Median follow-up time, months	27	18.2	10.8
Mean eGFR, mL/min/m^2^	68	66	61
eGFR < 60, %	37	41	53
NYHA class III–IV, %	25	33	41
ACE inhibitor/ARB, %	-	83.2	73.4
ARNI, %	-	10.8	14.5
Beta Blockers, %	93	96	93.1
MRA, %	60	71	70.3
ICD, %	15	26	27.8
CRT, %	7	7	14.7
HF Hospitalization < 6 months, %	31	16	84
Annualized Event Rate, n events per 100 patient-years at risk (placebo vs. treatment arm)	13.2 10.5	15.6 11.6	37.8 33.6
Primary Endpoint, HR (95% CI)	0.80 (0.73–0.87)	0.74 (0.65–0.85)	0.90 (0.82–0.98)
Absolute Rate Reduction, n	2.7	4	4.2

LVEF: left ventricular ejection fraction; NT-proBNP: NT-pro B-type natriuretic peptide; eGFR: estimated glomerular filtration rate; NYHA: New York Heart Association; ACE: Angiotensin-Converting Enzyme; ARB: Angiotensin Receptor Blocker; ARNI: Angiotensin Receptor Neprilysin Inhibitor; MRA: Mineralocorticoid Receptor Antagonist; ICD: Implantable Cardioverter Defibrillator; CRT: Cardiac Resynchronization Therapy; HR: Hazard Ratio; CI: Confidence Interval.

**Table 2:** Guideline recommendations for vericiguat in HFrEF.

Guideline	Year	Recommendation	Recommendation Strength	Evidence Quality
ESC/HFA [63]	2021	Vericiguat may be considered in patients in NYHA class II-IV who have had WHF despite treatment with an ACE-I (or ARNI), a beta-blocker and an MRA to reduce the risk of cardiovascular mortality or HF hospitalization.	IIb	B
CCS/CHFS [64]	2021	The authors recommend that vericiguat be considered in addition to optimal HF therapies for HFrEF patients with worsening symptoms and HF hospitalization in the past 6 months, to reduce the risk of subsequent HF hospitalization	Conditional Recommendation	Moderate-Quality Evidence
ACC/AHA/HFSA [65]	2022	In selected high-risk patients with HFrEF and recent WHF already on GDMT, vericiguat may be considered to reduce HF hospitalization and cardiovascular death.	IIb	B-R

HFrEF: heart failure with reduced ejection fraction; ESC: European Society of Cardiology; HFA: Heart Failure Association; CCS: Canadian Cardiovascular Society; CHFS: Canadian Heart Failure Society; ACC: American College of Cardiology; AHA: American Heart Association; HFSA: Heart Failure Society of America; NYHA: New York Heart Association; WHF: worsening heart failure; ACE-I: Angiotensin-Converting Enzyme Inhibitor; ARNI: Angiotensin Receptor Neprilysin Inhibitor; MRA: Mineralocorticoid Receptor Antagonist; HF: heart failure; GDMT: guideline-directed medical therapy. IIb recommendation strength: usefulness/efficacy is less well established by evidence/opinion. Evidence quality B: data derived from a single randomized clinical trial or large non-randomized studies. B-R: moderate-quality evidence from one or more randomized clinical trials or meta-analyses of moderate-quality randomized clinical trials

## Data Availability

Not applicable.

## Abbreviations

ACC	American College of Cardiology
ACE	Angiotensin-Converting Enzyme
AE	Adverse event
AHA	American Heart Association
ARB	Angiotensin Receptor Blocker
ARNI	Angiotensin Receptor Neprilysin Inhibitor
BNP	B-type natriuretic peptide
cGMP	Cyclic guanosine monophosphate
CAD	Coronary artery disease
CCS	Canadian Cardiovascular Society
CHFS	Canadian Heart Failure Society
CI	Confidence Interval
cNG	Cyclic nucleotide-gated
COPD	Chronic obstructive pulmonary disease
CRT	Cardiac Resynchronization Therapy
CYP	Cytochrome
DAPA-HF	Study to Evaluate the Effect of Dapagliflozin on the Incidence of Worsening Heart Failure or Cardiovascular Death in Patients with Chronic Heart Failure
eGFR	Estimated glomerular filtration rate
EMPEROR-Reduced	Empagliflozin Outcome Trial in Patients With Chronic Heart Failure with Reduced Ejection Fraction
eNOS	Endothelial nitric oxide synthase
ESC	European Society of Cardiology
FDA	Food and Drug Administration
GDMT	Guideline-directed medical therapy
HF	Heart failure
HFA	Heart Failure Association
HFrEF	Heart failure with reduced ejection fraction
HFSA	Heart Failure Society of America
HR	Hazard Ratio
KorAHF	Korean Acute Heart Failure
ICD	Implantable Cardioverter Defibrillator
LVEF	Left ventricular ejection fraction
MAGGIC	Meta-Analysis Global Group in Chronic Heart Failure
MRA	Mineralocorticoid Receptor Antagonist
NYHA	New York Heart Association
NO	Nitric oxide
NT-proBNP	N terminal pro B-type natriuretic peptide
PAH	Pulmonary arterial hypertension
PARADIGM-HF	Safety and Tolerability During Open-label Treatment with LCZ696 in Patients with CHF and Reduced Ejection Fraction
PDE	Phosphodiesterase
PINNACLE	Practice Innovation and Clinical Excellence
PKG	Cyclic GMP-dependent protein kinase
Q	Quartile
QoL	Quality of life
RAAS	Renin–Angiotensin–Aldosterone System
RCT	Randomized clinical trial
sGC	Soluble guanylate cyclase
SGLT2	Sodium–Glucose Transporter 2
SOCRATES-Reduced	Effect of Vericiguat, a Soluble Guanylate Cyclase Stimulator, on Natriuretic Peptide Levels in Patients with Worsening Chronic Heart Failure and Reduced Ejection Fraction
STRONG-HF	Safety: tolerability and efficacy of up-titration of guideline-directed medical therapies for acute heart failure
SwedeHF	Swedish Heart Failure
VICTOR	A Study of Vericiguat (MK-1242) in Participants with Chronic Heart Failure with Reduced Ejection Fraction (HFrEF) (MK-1242-035)
VICTORIA	Vericiguat Global Study in Subjects with Heart Failure with Reduced Ejection Fraction
WHF	Worsening heart failure
